# Mitochondrial Dysfunction, Mitophagy and Their Correlation with Perinatal Complications: Preeclampsia and Low Birth Weight

**DOI:** 10.3390/biomedicines10102539

**Published:** 2022-10-12

**Authors:** Raziye Melike Yildirim, Yagmur Ergun, Murat Basar

**Affiliations:** 1 Department of Obstetrics, Gynecology and Reproductive Sciences, Yale School of Medicine, New Haven, CT 06510, USA; 2 Yale Fertility Center, 200 West Campus Drive, Orange, CT 06477, USA

**Keywords:** preeclampsia, low birth weight, fetal growth restriction, mitophagy, mitochondria, perinatal complications

## Abstract

Mitochondria are essential organelles and crucial for cellular survival. Mitochondrial biogenesis and mitophagy are dynamic features that are essential for both maintaining the health of the mitochondrial network and cellular demands. The accumulation of damaged mitochondria has been shown to be related to a wide range of pathologies ranging from neurological to musculoskeletal. Mitophagy is the selective autophagy of mitochondria, eliminating dysfunctional mitochondria in cells by engulfment within double-membraned vesicles. Preeclampsia and low birth weight constitute prenatal complications during pregnancy and are leading causes of maternal and fetal mortality and morbidity. Both placental implantation and fetal growth require a large amount of energy, and a defect in the mitochondrial quality control mechanism may be responsible for the pathophysiology of these diseases. In this review, we compiled current studies investigating the role of BNIP3, DRAM1, and FUNDC1, mediators of receptor-mediated mitophagy, in the progression of preeclampsia and the role of mitophagy pathways in the pathophysiology of low birth weight. Recent studies have indicated that mitochondrial dysfunction and accumulation of reactive oxygen species are related to preeclampsia and low birth weight. However, due to the lack of studies in this field, the results are controversial. Therefore, mitophagy-related pathways associated with these pathologies still need to be elucidated. Mitophagy-related pathways are among the promising study targets that can reveal the pathophysiology behind preeclampsia and low birth weight.

## 1. Introduction

Mitochondria are essential organelles and are responsible for energy production for cellular survival. Therefore, dysfunctional or damaged mitochondria are related to a wide spectrum of pathologies, especially in tissues with the highest energy requirements [1]. Because mitochondria are crucial for the maintenance of the energy level of cells and for cellular survival, mitochondrial homeostasis is strictly regulated by mitochondrial biogenesis and mitophagy [2,3,4]. Mitophagy is the selective elimination process of damaged mitochondria by engulfment, and several molecular pathways are required for properly functioning mitophagy [5]. Preeclampsia and low birth weight constitute prenatal complications and are among the leading causes of maternal and fetal mortality and morbidity [6,7,8]. The placenta is a highly energy-dependent organ due to its crucial role as an exchange interface for metabolites, as well as its endocrinal function for the maintenance of pregnancy. It is also the main predictor of pregnancy outcomes, and malfunctioning of the placenta can result in severe diseases, such as preeclampsia and low birth weight [9,10,11]. Therefore, it is crucial to obtain a more comprehensive understanding of how placental mitochondria orchestrate changes in connection to pregnancy outcomes.

There are still major milestones in the field of mitophagy to be achieved. In this review, we compiled studies that investigated the effects of key components of mitochondrial dysfunction and mitophagy on the pathophysiology of preeclampsia and low birth weight.

## 2. Mitochondrial Homeostasis

**Figure 1:**
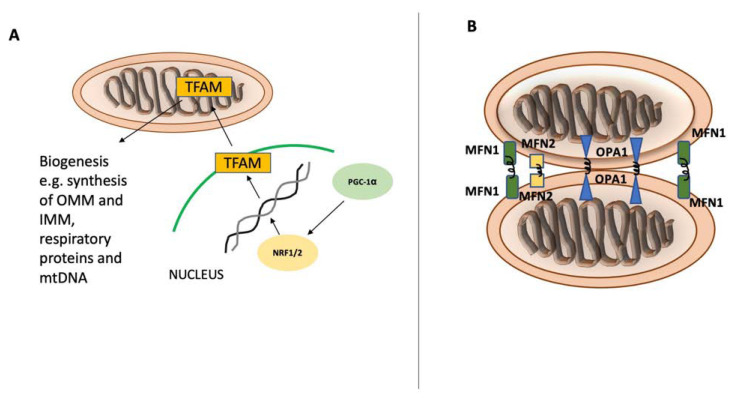
Mitochondrial biogenesis and fusion. Mitochondrial biogenesis and fusion. Peroxisome proliferator-activated receptor gamma coactivator 1-alpha (PGC-1α) is the main regulator of mitochondrial biogenesis (**A**) and translocates into the nucleus after its activation. In the nucleus, PGC-1α stimulates NRF1 and NRF2 expression, leading to increased activity of mitochondrial transcription factor A (TFAM), which orchestrates the synthesis of mtDNA and proteins. Mitochondrial fusion (**B**) is controlled by mitofusin-1 (MFN1) and mitofusin-2 (MFN2) on the mitochondrial outer membrane and by optic atrophy 1 (OPA1) on the inner mitochondrial membrane.

Mitochondria are essential organelles in cellular metabolism and physiology. Defective mitochondria are related to a broad spectrum of pathologies ranging from neurological to musculoskeletal [1]. Mitochondrial biogenesis and autophagy of mitochondria are two main pathways that preserve mitochondrial content and quality in cells. The tight regulation of these opposing pathways enables cells to adapt to the cellular energy demand and to respond to both intracellular and extracellular stress [12]. Mitochondria have their own circular genome that encodes 13 essential proteins for respiratory complexes. However, most mitochondrial proteins are encoded by nuclear DNA synthesized within the cytosol and imported into mitochondria. Several transcription factors are activated to control mitochondrial biogenesis in response to a variety of stimuli, including nutrition availability, hormones, growth factors, and temperature changes. Nuclear respiratory factors (NRF1 and NRF2), estrogen-related receptors (ERR-α, ERR-β, and ERR-γ), and the peroxisome proliferator-activated receptor gamma coactivator 1-alpha (PGC-1α) are among the key regulators of mitochondrial biogenesis. NRF1 and NRF2 drive the expression of the necessary nuclear genes encoding the proteins related to mitochondrial functioning. Additionally, transcription factor A (TFAM) and transcription factor B (TFB) proteins are important regulators of mitochondrial DNA transcription and replication and are regulated by both NRF1 and NRF2 [3,12,13] (Figure 1). Another group of nuclear hormone receptors including estrogen-related receptors ERR-α, ERR-β, and ERR-γ can stimulate mitochondrial biogenesis. They mainly regulate the transcription of genes involved in Krebs’ cycle, fatty acid oxidation, mitochondrial fusion, and fission. Furthermore, PGC-1α, a member of peroxisome proliferator-activated receptors (PPARs), serves as a coactivator and governs the activity of a wide range of transcription factors related to mitochondrial proliferation [4,12].

As damaged mitochondria can be detrimental to cellular homeostasis, several quality control mechanisms have evolved to ensure the smooth functioning of cells, including the production of adenosine triphosphate (ATP) [14]. Mitochondrial fusion is the joining of two mitochondria through their outer and inner membrane interfaces via membrane GTPases called mitofusin-1 (MFN1), mitofusin-2 (MFN2), and optic atrophy protein-1 (OPA1) (Figure 1). This mechanism allows dysfunctional mitochondria to exchange metabolites and proteins to cope with oxidative stress and enhance their overall respiratory function [15]. However, mitochondrial fusion is a rescue mechanism and is selective for polarized and active mitochondria. If mitochondria are severely damaged or depolarized, they become the target of autophagy for digestion and elimination [16]. Autophagy is an evolutionarily conserved quality control mechanism by which sequestered cytoplasmic components and organelles are packaged in double-membrane vesicles to be transported to the lysosome for degradation. This pathway enables cells to eliminate unwanted, dangerous, and damaged components, including dysfunctional mitochondria. Depending on the component to be degraded, autophagy can be selective or non-selective. In non-selective autophagy, the phagophore sequesters large sections of the cytoplasm for degradation. This kind of autophagy typically occurs during starvation with low nutritional levels. However, selective autophagy is a result of the autophagosome’s evolutionary adaptation to tackle the number and integrity of cellular organelles, such as ER, ribosomes, peroxisomes, and mitochondria. Selective autophagy receptors (SARs) are necessary for the identification of cargo and attachment of the cargo to the phagophore. Protein alterations, primarily phosphorylation, ubiquitination, acetylation, and oligomerization, control the activity of SARs [17,18,19]. Mitophagy is termed as selective destruction of damaged mitochondria, during which a portion of the mitochondrial network is gradually engulfed within a double-membraned autophagosome called mitophagosome. It is a process that involves multiple molecular pathways and is strictly regulated.

**Figure 2:**
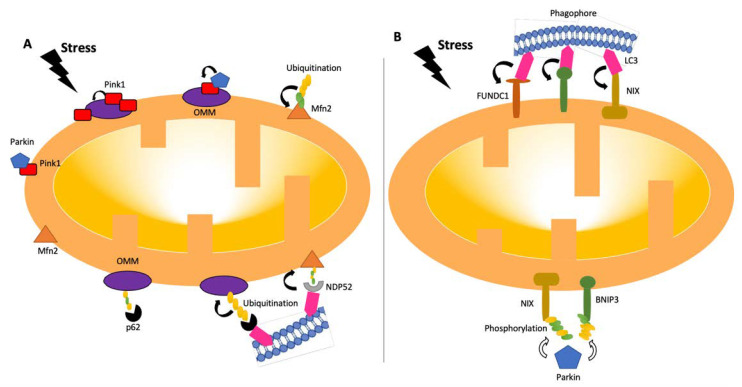
Pathways of mitophagy. Mitophagy pathways are either receptor- (**B**) or ubiquitin- (**A**) dependent [24,26]. Under stress conditions, mitochondria become dysfunctional, and PINK1 accumulates and stabilizes on the outer membrane of mitochondria, triggering PARKIN activation [27]. PARKIN recruits the ubiquitination and phosphorylation processes of outer-membrane proteins. Adaptor proteins, such as p62 and NDP52, recognize phosphorylated poly-ubiquitin chains. LC3 connects to adaptor proteins, and phagophores recognize LC3. In the receptor-mediated pathway (**B**), LC3 attaches to FUNDC1, BNIP3, or NIX receptors. BNIP3 or NIX can also be phosphorylated (PARKIN-mediated or -independent). LC3: microtubule-associated protein 1A/1B-light chain 3; BNIP3: Bcl-2/adenovirus E1B 19-kDa-interacting protein 3; FUNDC1: mitochondrial receptor FUN14 domain-containing 1; NIX: BCL2/adenovirus E1B 19 kDa protein-interacting protein 3-like.

In order to facilitate mitophagy, certain prerequisites must be met following mitochondrial damage, such as fission of the mitochondrial network, priming of the mitochondrion for recognition, and engulfment of the mitochondrion via the formation of autophagosome [5]. The GTPase dynamin-related protein 1 (Drp1) catalyzes mitochondrial fission, which involves splitting of the mitochondrial network into smaller pieces so mitochondria can be accommodated in the phagosome. Several adaptor proteins found on the surface of the mitochondria recruit Drp1 from cytosol at locations where endoplasmic reticulum (ER) tubules encounter mitochondria. This is followed by Drp1 oligomerization and GTP hydrolysis, which leads membrane constriction at fission sites [5,20]. It is also essential to understand how mitochondrial fission and mitophagy are coupled. PTEN-induced putative kinase 1 (PINK1), a critical mitophagy-initiating kinase, has been shown to alleviate the inhibitory effects of protein kinase A (PKA) on Drp1 phosphorylation and to promote fission [21]. Another key mitophagy receptor, FUN14 domain-containing 1 (FUNDC1), stimulates fission by direct association with Drp1 [22]. Cells possess multiple mitophagy mechanisms (Figure 2), and various stimuli can promote mitophagy via multiple signaling cascades in different cellular contexts [23].

At times of mitochondrial malfunctioning, mitophagy eliminates dysfunctional mitochondria and maintains the population in a healthy state. Cells have developed many frequently overlapping processes to ensure that mitophagy can proceed in a balanced manner in response to a variety of stimuli and triggers because the precise and timely removal of mitochondria appears to be vital for cell survival [2]. Mitophagy regulatory pathways can be classified as ubiquitin-dependent or -independent. However, studies have revealed that crosstalk occurs between these signaling pathways [23,24].

In the ubiquitin-dependent pathway, poly-ubiquitin-tagged outer mitochondrial membrane components are required for recognition by autophagic machinery. Among the regulators of ubiquitin-dependent mitophagy are the PINK1-Parkin pathways. These two major proteins play crucial roles in damage-induced mitophagy [25,26,27]. Under stable conditions, PTEN-induced putative kinase 1 (PINK1) is constantly transported from the outer mitochondrial membrane (OMM) to the inner mitochondrial membrane (IMM) and cleaved by several proteases in functional mitochondria. This process allows the cellular levels of PINK1 to remain low [28,29]. Following the incident leading to potential mitochondrial membrane disruption, PINK1 acts as a molecular sensor of mitochondrial damage. The transportation of PINK1 is prevented due to mitochondrial membrane depolarization and causes the accumulation of PINK1 on OMM. Parkin is translocated to the mitochondrial surface as a result of PINK1 stabilization on OMM and its activation by autophosphorylation. The full activation of Parkin by PINK1-mediated phosphorylation at the respective serine 65 residues leads to conformational changes in the intramolecular structure of Parkin. Then, Parkin promotes elongation of pre-existing ubiquitin chains on OMM, which also serve as PINK1 substrates [30,31]. Parkin facilitates a feed-forward mechanism to produce poly-Ub chains, boosting mitophagy signals [2,29,32]. Phosphorylated poly-Ub chains on mitochondrial proteins are recognized by adaptor proteins (p62, OPTN, and NDP52 (nuclear dot protein 52)), which then bind to LC3 to initiate the production of autophagosomes [2]. Along with Parkin, several additional ubiquitin E3 ligases, including Gp78, SMURF1, SIAH1, MUL1, and ARIH1, are involved in the control of mitophagy. After localization on the mitochondrial outer membrane, they produce ubiquitin chains, which cause the recruitment of certain autophagy adaptors, such as optineurin (OPTN), nuclear dot protein 52 (NDP52), and p62, among others [2,33,34]. Once adaptor proteins are attached, they directly interact with light chain 3 (LC3), anchoring ubiquitinated mitochondria to autophagosomes [2].

Besides ubiquitin-dependent pathways, various mitochondrial proteins, including NIP-3 like protein X (NIX), FUNDC1 (FUN14 domain containing 1), and BNIP3 also serve as mitophagy receptors and direct defective mitochondria to autophagosomes for elimination. Those mitophagy receptors directly engage with LC3 through their LC3 interaction region (LIR) motifs and provide another mechanism for the destruction of dysfunctional mitochondria [35]. Whereas NIX plays a crucial role in programmed mitophagy during differentiation, BNIP3 alters the dynamics of the mitochondria, leading to the splitting of damaged mitochondria by causing OPA1 to disassemble and release and DDRP1 to be recruited to the mitochondrial surface [36]. Through control of Parkin recruitment, NIX and BNIP3 maintain mitochondrial homeostasis, indicating crosstalk between mitophagy receptors and the PINK1-Parkin pathway [37].

## 3. Insights into Preeclampsia

Preeclampsia (PE) affects 3% to 7% of pregnancies [7] and is one of the leading causes of maternal and fetal/neonatal morbidity and mortality [38,39]. The most common symptoms are the presence of both hypertension (systolic BP 140 mm Hg and/or diastolic BP 90 mm Hg) and proteinuria (0.3 g/24 h), both of which emerge after 20 weeks of gestation. Patients can also present without proteinuria but experience hypertension, along with characteristics including elevated liver enzyme activities, increased creatinine, seizures, thrombocytopenia, or intrauterine growth restriction [6]. Early-onset PE (type I) manifests before 34 weeks of gestation, whereas late-onset PE (type II) presents after this time point. The only known treatment for either type of PE is delivery of the placenta [40]. Although the pathophysiology of PE remains poorly understood, PE may develop as a result of an imbalance between increased oxidative stress and antioxidative defense mechanisms. Although pro-oxidants and reactive oxygen species (ROS) are produced at higher levels during the later stages of a healthy, uncomplicated pregnancy, these are effectively controlled by the buildup of antioxidants, such as superoxide dismutase (SOD), glutathione (GSH), tocopherols, carotenoids, and ascorbic acid [41]. However, in PE, this balance between pro-oxidants and antioxidants is disturbed [42]. Although multiple ROS sources in the placenta have been identified [43], such as decreased antioxidant activity and a lack of blood flow, few studies have investigated the role of mitochondrial dysfunction and ROS in the pathology of PE. Mitochondrial dysfunction reduces oxygen consumption and increased superoxide production [44].

## 4. Preeclampsia and Mitochondria

The placenta plays a crucial role in the constitution of maternal and fetal, in addition to its endocrinal function for the continuation of pregnancy and fetal well-being. Owing to its wide range of functions, the placenta is a highly active organ that depends on functioning mitochondria for energy [45]. Mitochondria are dynamic organelles with their own metabolism, constantly undergoing fusion and fission according to cellular needs and changes. Mitochondria also represent the main energy source enabling placental implantation and development. It has been shown that shallow placental implantation and improper spinal artery remodeling lead to ischemia and an increase in ROS, which are core risk factors for PE development [46,47]. Both excessive levels of ROS production and impaired antioxidant defense have been reported in preeclamptic placental samples. Moreover, expression levels of various antioxidant enzymes were reported to suppressed in PE placentae [48]. According to a study investigating the effects of mitochondrial dysfunction on placental metabolism by comparing detailed metabolomic pathway analysis of PE and healthy placenta, 21 metabolic pathways were significantly downregulated in PE placentae. In particular, four of the identified pathways were associated with lipid metabolism, demonstrating that the ATP levels and enzymes responsible for fatty acid catabolism were reduced in PE [49]. Mitochondrial fusion enables dysfunctional mitochondria to replenish their cellular content by replacing healthier mitochondrion, whereas fission occur when mitochondria are damaged or exposed to high levels of stress [50,51]. Zhou et al. analyzed the expression levels of fusion-responsible proteins mitofusin-1 (MFN1), mitofusin-2 (MFN2), and optic atrophy 1 (OPA1). Results indicated a significant decrease in fusion-related proteins in PE placentae compared to healthy placentae, whereas no statistical differences were observed in fission-related proteins of DRP1 and Fis1 levels in PE [49]. In another study specifically exploring the association between MFN2 expression and PE, Yu et al. first measured ATP levels to prove mitochondrial dysfunction. Their results showed that both ATP and MFN2 expression levels were reduced compared to controls. Moreover, they tested the gradual hypoxia conditions by one cultured trophoblast and measured MFN2 expression levels. Results showed significantly downregulated MFN2 levels correlated with the degree of hypoxia [52]. In contrast to these findings, Ausman et al. examined PE placentae and found significantly higher levels of DRP1. They also reported that mitochondrial fission was accompanied by an increase in ceramides (CERs) in PE placentae relative to controls. CERs are inducers of intrinsic cellular death mechanism-involving BOK, a proapoptotic member of the Bcl-2 family that also triggers MFN2 to endoplasmic reticulum tethering to facilitate the progression of mitochondrial fission. When Ausman et al. analyzed transmission electron microscopy images of PE placentae, results showed significantly increased mitochondrial fission in close relation to the endoplasmic reticulum, which is also supported by upregulated MFN2 levels of samples collected from PE placentae relative to controls [53]. Mitochondrial DNA turnover among the crucial parts of mitochondrial biogenesis. Its expression is upregulated by the activity of NRF1 and TRAM genes. Analyzed expression levels of those genes in placental samples indicate that TFAM levels are 1.8-fold lower in PE compared to controls; however no significant differences were observed in terms of expression levels of NRF1. The same study also investigated the mtDNA copy number in PE placental samples and, showing that mtDNA copy number is significantly lower in early-onset PE, whereas there is no difference between late-onset PE and controls [54]. The results of another study on preeclamptic myometrium implied no significant change in mtDNA copy number but an increase in TRAM expression in PE [55]. All these studies indicate that data obtained with respect to the expression levels of genes responsible for mitochondrial fusion, fission, and biogenesis are controversial; therefore, further research is required in this field to improve our understanding.

## 5. Markers of Mitophagy and Effects on Preeclampsia

Because mitophagy basically constitutes the elimination of dysfunctional mitochondria and provision of cells with appropriate energy metabolism according to their needs, any defect in the mitophagy pathway may be responsible for the increment of cellular ROS and, eventually, the development of preeclampsia. Recent studies on the mitophagy-responsible proteins discussed below have shown that they are associated with preeclampsia, requiring future investigations.

### 5.1. BNIP3

Bcl-2/adenovirus E1B 19-kDa-interacting protein 3 (BNIP3) is a Bcl-2 family pro-apoptotic mitochondrial protein that is found in a variety of human organs, including the placenta [56]. Hypoxia-induced cell death is regulated by BNIP3, which interacts directly with the microtubule-associated protein 1A/1B-light chain 3 (LC3) or recruits Parkin [23]. In a state of hypoxia or low energy, BNIP3 prevents PINK1 from degrading, instead causing its accumulation on the mitochondrial outer membrane. PINK1 phosphorylates both Parkin and ubiquitin. Phospho-ubiquitin interacts with LC3II-coated phagophore trough p62. BNIP3 can also directly interact with LC3II and mediate the interactions of phagophore [10]. According to a study by Zhou et al., BNIP3 expression was suppressed, along with impaired autophagy and accumulation of damaged mitochondria, in PE placentae. Scanning electron microscopy showed that dilated endoplasmic reticulum and swollen mitochondria were accumulated in PE placentae, also proving insufficient autophagic activity necessary to achieve mitochondrial recycling. As a part of the abovementioned study, the authors successfully silenced BNIP3 and investigated the expression levels of p62, Beclin-1, and PINK1 to determine the influence of BNIP3 on autophagosome formation. They found that downregulation of BNIP3 significantly inhibited the expression of Beclin-1 but increased the expression of p62. Interestingly, it caused no significant changes in PINK1 levels. The authors also reported that suppression of BNIP expression inhibited autophagosome formation and mitophagy by visualizing colocalization of mitochondria and lysosome while promoting ROS accumulation and apoptosis in oxidative stress. Moreover, through proliferation assay, the authors found that the downregulation of BNIP3 caused attenuation of invasion and migration capacity, although there was no influence on cell proliferation [10]. Tong et al. conducted an RNA-Seq analysis to compare gene expression in placentae from early PE, late PE, and healthy subjects and found 293 PE-related genes, confirming the downregulation of BNIP3 in PE placentae [57]. Research investigating the influence of BNIP3 on decidua and preeclampsia by Ma et al. demonstrated that preeclampsia pregnancies were associated with lower levels of BNIP3 mRNA and protein in the decidua relative to normal pregnancies. The transcription of PRL and IGFBP-1, which are endometrial decidual markers, was significantly reduced as a result of BNIP3 knockdown, which also blocked morphological alterations after the induction of decidualization and might be responsible for the PE phenotype [58]. Vangrieken et al. comprehensively assessed the indices of mitochondrial biogenesis and mitophagy in preeclamptic (*n* = 12) and control (*n* = 11) placentae. In PE placentae, protein levels of BNIP3 and BNIP3L were significantly higher, whereas PINK1, Parkin, and FUNDC1 levels were similar to those of the controls. Surprisingly, mRNA expression levels of those mitophagy related proteins were unchanged in PE placentae, except for the FUNDC1 transcription levels. The also indicated that receptor mediated mitophagy pathways might be involved in the pathology of PE [59]. In contrast to the results reported by Vangrieken et. al, Ausman et al. conducted a study revealing increased levels of PINK1, non-cleaved, and Parkin in PE placentae compared to controls. This suggests the possibility of a Parkin-dependent pathway that may be primed in PE, whereby if mitochondrial dysfunction exists, Pink1 is not cleaved properly and accumulates on the mitochondrial outer membrane, recruiting Parkin and activating mitophagy [53].

### 5.2. FUNDC1

Mitochondrial receptor FUN14 domain-containing 1 (FUNDC1) is a mitochondrial autophagy receptor expressed in the outer membrane of the mitochondria that mediates hypoxia-induced mitophagy. By directly binding to the LC3 (mammalian Atg8 homologue), it activates mitophagy [60]. Normally, FUNDC1 mitophagy receptor activity is regulated through inhibition of phosphorylation by Src kinase. However, under hypoxic conditions, dephosphorylation of FUNDC1 strengthens its interaction with light chain 3 (LC3), eventually inducing the formation of mitophagosomes [61]. In a recent study comparing FUNDC1 ubiquitination levels in placental tissues of normal pregnant women and in placental tissues of PE patients, PE patients were reported to have lower levels of FUNDC1. Furthermore, the authors reported that enhanced FUNDC1 ubiquitination could alleviate trophoblast cell injury in a mouse model of PE [62]. Another study showed that mRNA expression levels of FUNDC1 were lower in the PE group compared to controls [59].

### 5.3. DRAM1

Transmembrane protein family member DNA damage-regulated autophagy modulator 1 (DRAM1) has been demonstrated to be essential for TP53-dependent autophagy activation and apoptosis. [63]. According to a study by Zhang et al., it is specifically responsible for autophagy activation induced by mitochondrial dysfunction [64]. Mice were induced by Hif-1 alpha to create a PE mouse model in a recent study by Chen et al. to examine the response of DRAM1 toward mitochondrial dysfunction. Serial measures of blood pressure, TG, HDL, LDL, and urine protein were performed. In contrast to the wild type, the Hif-1 alpha-induced group had significantly higher levels of blood pressure, LDL, and urine protein. The authors reported higher levels of H_2_O_2_ and MDA, a naturally occurring byproduct of lipid oxidation, and lower levels of SOD (superoxide dismutase), an antioxidant enzyme in the placentae of PE mouse models. Additionally, they investigated the expression of cytochrome c oxidase IV (COX IV), one of the major proteins of the electron transport chain in mitochondria and found that it was much lower in their mouse model than in wild-type mice. To detect mitophagy activity, they determined the protein expression of PINK1 and Parkin which are responsible for the initiation of mitophagy, and DRAM1, which plays a significant role in autophagy. According to the findings, PINK1 and DRAM1 levels were considerably lower than those of the wild type. To learn more about the function of DRAM1 in PE, researchers introduced a DRAM1 overexpression plasmid into the trophoblasts of PE mouse placentae. They discovered that DRAM1 overexpression significantly reduced MDA levels and improved TG and urinary protein levels in PE mice but did not significantly alter H_2_O_2_ levels. The effects of DRAM1 overexpression on mitophagy were also assessed, and PINK1 and Parkin protein expressions were found to be increased significantly compared to the control group [65]. Fusion and fission, which are both governed by a family of GTPases, are two mechanisms by which mitochondrial dynamics are controlled by mitochondrial biogenetics. MFN1/2 and OPA1 are needed for fusion, whereas DRP1 mediates fission. [66]. The study also showed that DRAM1 overexpression significantly increased fusion-related protein expression. Together with all these findings, Chen et al. [65] concluded that overexpression of DRAM1 can promote mitophagy and enhance mitochondrial function. Zhang et al. also found that MFN2 expression was downregulated in PE placentae, which is consistent with these results [67].

## 6. Intrauterine Growth Restriction (IUGR) and Low Birth Weight

Fetal growth restriction (FGR), formerly known as intrauterine growth restriction (IUGR), is a key factor in prenatal and neonatal morbidity and mortality and considered a disease that can develop as a result of several factors [8]. It is defined as a below-average fetal growth rate compared to the potential for growth of a newborn. Asymmetrical IUGR (malnourished infants), symmetrical IUGR (hypoplastically small for date), and mixed IUGR are the three main kinds of IUGR based on several clinical and anthropometric characteristics [68]. Maternal, placental, fetal, or genetic factors frequently lead to IUGR, although any of these variables alone or in combination can cause IUGR. IUGR can also result from differences between the placenta’s supply of nutrients and the fetus’s demand for those nutrients. IUGR is occasionally caused by fetal deformities, inborn metabolic errors, and chromosomal abnormalities. The function of numerous maternal, fetal, and placental gene polymorphisms has increased in significance as a result of recent developments in molecular biology and genetics and are now recognized as a possible cause of IUGR [69,70,71]. According to two systematic analyses, the average global low birth weight ratio is 14.6% [72,73].

Low birth weight (LBW) is defined by the World Health Organization as any birth weight that is less than 2500 g, regardless of gestational age [74]. Compared to neonates of normal weight, LBW infants are 20 times more likely to experience problems and die [3]. LBW infants are at-risk of developing cognitive impairments, motor delays, cerebral palsy, and behavioral and psychiatric issues [75,76]. Methods to decrease the incidence of low birth weight and premature birth may have a noticeable impact on newborn infection. Delaying childbearing in adolescents, improving maternal education, caloric supplementation before and during pregnancy, general improvements in nutrition, malaria prophylaxis or treatment, treatment of other maternal infections, efforts to reduce tobacco use, limiting maternal work during pregnancy, and general improvements in society are potential interventions that could increase intrauterine growth, lengthen gestation, or both. The pathophysiology of low birth weight is unknown; however, preterm birth and intrauterine growth restriction (IUGR) are thought to be its causes [77].

Despite additional maternal symptoms of the condition, fetal IUGR has historically been part of the diagnostic standards for severe preeclampsia. Preeclampsia has traditionally been assumed to cause some of the worst cases of IUGR.

Additionally, preeclampsia is connected to a fourfold increase in the chance of IUGR, which has both immediate and long-term health effects [78]. Those who have IUGR have an increased chance of developing diabetes, obesity, hypertension, and cardiovascular disease later in life. According to a study published by Surico et al. [79], human mesenchymal stem cells (hUMSCs) from patients with PE and IUGR have distinct characteristics relative to those from normal pregnancies. Furthermore, the etiopathology of IUGR and PE caused by oxidative stress may be influenced by the interaction between hUMSCs and trophoblast cells. Both preeclampsia and IUGR are often characterized by poor placentation, which results in insufficient uteroplacental blood perfusion and ischemia, even if the exact causes of these conditions remain unknown.

## 7. Mitochondria and Its Association with Low-Birth-Weight Rate

**Figure 3:**
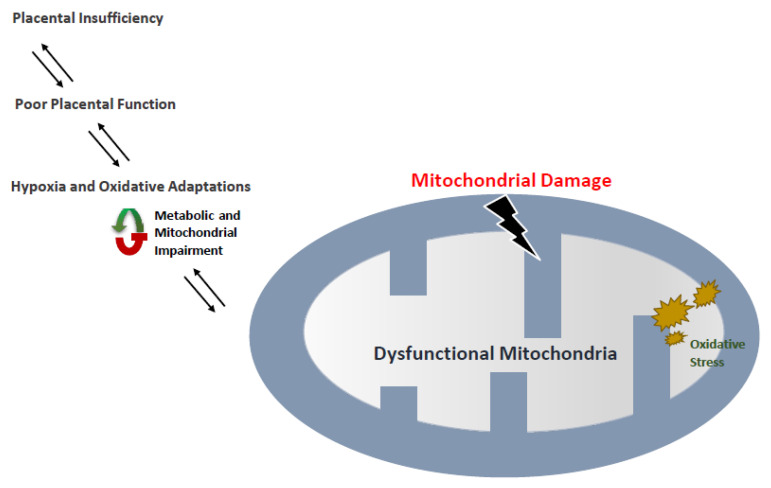
Mitochondria and IUGR. Insufficient placental growth results in IUGR, which causes metabolic and mitochondrial rearrangements by creating a hypoxic environment during fetal development. In these circumstances, the metabolic and mitochondrial imbalance can eventually result in the development of IUGR under oxidative stress conditions.

Both macro- and micronutrients are necessary to support fetal development and the nutritional demands of pregnancy [80]. According to previous studies, intrauterine growth restriction (IUGR), placental abruption, and reduced birth weight are possible outcomes of increased mtDNA copy number in maternal blood. Alterations in mtDNA copy number can lead to decreased efficiency of the electron transport chain (ETC) mechanisms and oxidative phosphorylation system (OXPHOS), as well as increased ROS production [81] (Figure 3). Priliani et al. studied the effects of increased mtDNA copy number in maternal peripheral blood in Indonesia and found that venous blood mtDNA copy number was a significant predictor of IUGR and birth weight (BW) [80]. According to their study, the mtDNA copy number assessed from cord blood, placental, and maternal peripheral blood was inversely correlated with low BW and was found to be a possibly useful marker of risk for IUGR. Lattuada et al. evaluated placental samples collected from 50 singleton pregnancies and found that mtDNA copy number was differed significantly between IUGR and normal intrauterine growth samples [82]. Furthermore, their findings suggested an increase in mtDNA copy number was inversely related to oxygen tension in the umbilical vein. These findings suggest that when the ATP is required to maintain the transport of nutrients across the maternal–fetal transition, ETC mechanisms regulate mitochondrial functionality [83]. Because mitophagy controls the quality of the mitochondria by eliminating dysfunctional mitochondria [84], absent or deficient mitophagy are among the possible causes of mitochondria-related diseases.

## 8. Fetal Growth Restriction and Correlation with Mitophagy

As discussed above, FGR can be caused by several factors. Epigenetics are another possible cause of FGR. Zhu et al. designed a study to investigate whether cadmium (Cd) environmental toxicant and exposure induces the restriction of fetal growth by triggering PERK-regulated mitophagy in placental trophoblasts [85]. They used Cd as an environmental stressor to stimulate FGR in pregnant mice. Their data suggested that exposure to Cd caused a reduction in progesterone (P4) levels in placentae and amniotic fluids in mice, resulting in intrauterine growth restriction. Additionally, Cd treatment suppressed the regulation of the cholesterol side-chain cleavage enzyme gene (CYP11A1), which is a crucial P4 synthase, and triggered BNIP3-dependent mitophagy in placental trophoblasts by blocking the mechanisms of a serine/threonine-protein kinase general control nonderepressible 2 (GCN2), activating transcription factor 4 (ATF4). Correspondingly, it has been shown that mitophagy was also triggered by degradation of histone protein 60 (HSP60) and cytochrome C oxidase subunit 4 (COX4) under the effect of Cd in human placental trophoblasts [86]. Although few studies have been conducted related to human IUGR pregnancies and mitochondria and the results thereof are contradictory, some studies have reported on the mitochondrial implications in placenta via metabolomic and transcriptomic analysis in IUGR infant sera and mtDNA copy number disarrangement in cytotrophoblast cells [11,87,88,89].

## 9. Conclusions and Future Directions

Most of chronic or metabolic diseases are caused by a loss of function in mitochondria, the organelles responsible for cellular energy production. The following modifications lead to an alteration and impairment in mitochondrial function at the molecular level: (1) a reduction in the transfer of essential metabolites into mitochondria; (2) changes in the maintenance process of the electron transport chain, or (3) a loss of maintenance of the electrical and chemical transmembrane potential of the inner mitochondrial membrane. As a result of these modifications, oxidative phosphorylation is less effective, and less adenosine-5’-triphosphate is produced (ATP) [90]. Fission, i.e., the creation of entirely new mitochondria, the removal and complete degradation of dysfunctional mitochondria (mitophagy), the fusion of partially dysfunctional mitochondria, and the mixing of their healthy component can all alter the number and functional status of mitochondria cells [16]. Throughout pregnancy, mitochondria, which play key roles in cells, drive placental physiology, including placental development, nutrient exchange, and hormone release. Modifications of mitochondrial function may help to mediate or decrease the effects of adverse gestational settings on placental physiology and, consequently, the risks of pregnancy problems. Developmentally, placental mitochondria adapt to increasing fetal demands. In all mammals, the placental energy requirement corresponds primarily to OXPHOS [91]. Although placental mitochondria adapt developmentally and in response to hypoxia to support fetal growth [92], many unknowns remain with respect to gestational changes in placental oxidative stress and expression of mitochondrial-related proteins in several species. Although impaired mitochondrial fusion, along with autophagy and lipid metabolism deficiencies, have been associated with preeclampsia in many studies [49,53], the relation between probable causes of defective elimination of dysfunctional mitochondria and preeclampsia remains to be elucidated. Furthermore, various animal models of IUGR summarize pathophysiological conditions found in human fetuses with IUGR. In their role as the major metabolic organelle of cells, mitochondria regulate cellular metabolism by coordinating oxygen uptake with substrate usage, along with tissue energy accumulation and demand [14]. It is essential to decrease mitochondrial metabolic capacity in IUGR babies in response to nutritional limitations to ensure fetal survival. However, if these adaptations are long-lasting, IUGR fetuses may be predisposed to metabolic problems for the rest of their lives. The association of mitochondria and the energy production mechanism has been studied by many researchers, although the connection between mitophagy and IUGR requires further investigation.

The abovementioned studies demonstrate that further research is required to reveal the mechanism of mitophagy orchestration and its association with perinatal pathologies. The molecular pathways responsible for the elimination of dysfunctional mitochondria might play a key role in preeclampsia and low birth weight development. Thus, the use of animal disease models and studies on human placental samples could facilitate understanding of the relationship between mitophagy and these pathologies. Future investigations of mitophagy-regulating molecules might provide new perspectives on disease prevention and treatment strategies.

## Data Availability

Not applicable.

## Abbreviations

ATF4	Activating transcription factor 4
ATP	Adenosine Triphosphate
BNIP3	Bcl-2/adenovirus E1B 19-kDa interacting protein 3
BP	Blood pressure
CERs	Ceramides
CYP11A1	Cholesterol side-chain cleavage enzyme gene
COX IV	Cytochrome c oxidase IV
COX4	Cytochrome C Oxidase Subunit 4
DRAM1	DNA damage-regulated autophagy modulator 1
Drp1	Dynamin-related protein 1
ETC	Electron Transport Chain
ER	Endoplasmic Reticulum
ERR-α, ERR-β, ERR-γ	Estrogen-related Receptors -α,-β,-γ
FUNDC1	FUN14 domain containing 1
GCN2	General control nonderepressible 2
GSH	Glutathione
HSP6	Histone Protein 60
hUMSCs	Human Mesenchymal Stem Cells
IMM	Inner Mitochondrial Membrane
IUGR	Intrauterine growth restriction
LIR	LC3 interaction region
LC3	Light Chain 3
LBW	Low Birth Weight
mtDNA	Mitochondrial DNA
TFAM	Mitochondrial Transcription Factor A
MFN-1	Mitofusin-1
MFN-2	Mitofusin-2
NIX	NIP-3 like protein X
NDP52	Nuclear dot protein 52
NRF1	Nuclear respiratory factor 1
NRF2	Nuclear respiratory factor 2
OPA1	Optic Atrophy 1
OPTN	Optineurin
OMM	Outer Mitochondrial Membrane
OXPHOS	Oxidative phosphorylation system
PGC-1α	Peroxisome proliferator-activated receptor gamma co-activator 1-alpha
PE	Preeclampsia
PKA	Protein kinase A
PINK1	PTEN-induced putative kinase 1
ROS	Reactive oxygen species
SOD	Superoxide dismutase
TFB	Transcription Factor B
